# Daidzein modulates cocaine-reinforcing effects and cue-induced cocaine reinstatement in CD-1 male mice

**DOI:** 10.1007/s00213-021-05820-z

**Published:** 2021-04-11

**Authors:** Miquel Martin, Miriam Gutiérrez-Martos, Roberto Cabrera, Klaus Langohr, Rafael Maldonado, Magi Farre, Rafael de la Torre

**Affiliations:** 1 Integrative Pharmacology and Systems Neuroscience Research Group, Hospital del Mar Medical Research Institute (IMIM), Parc de Recerca Biomedica de Barcelona (PRBB), C/Dr. Aiguader 88, 08003 Barcelona, Spain; 2 Laboratory of Neuropharmacology, Parc de Recerca Biomedica de Barcelona (PRBB), Universitat Pompeu Fabra, C/Dr. Aiguader 88, 08003 Barcelona, Spain; 3 Department of Statistics and Operations Research, Universitat Politècnica de Cataluña (UPC)/BarcelonaTech, Jordi Girona 1-3, 08034 Barcelona, Spain; 4 Universitat Pompeu Fabra (CEXS-UPF), C/Dr. Aiguader 88, 08003 Barcelona, Spain; 5 Universitat Autònoma de Barcelona (UDIMAS-UAB), C/Dr. Aiguader 88, 08003 Barcelona, Spain; 6 Clinical Pharmacology Unit, Hospital Universitari Germans Trias i Pujol (IGTP), Carretera de Canyet s/n, 08916 Badalona, Spain; 7 CIBER Fisiopatologia de la Obesidad y la Nutrición (CIBERobn), Choupana s, /n 15706 Santiago de Compostela, Spain

**Keywords:** cocaine, daidzein, daidzin, dopamine receptors, genistein, isoflavones, reinstatement, reinforcing, self-administration

## Abstract

**Rationale:**

Cocaine addiction is a chronic relapsing disorder that lacks of an effective treatment. Isoflavones are a family of compounds present in different plants and vegetables like soybeans that share a common chemical structure. Previous studies have described that synthetic derivatives from the natural isoflavone daidzin can modulate cocaine addiction, by a mechanism suggested to involve aldehyde-dehydrogenase (ALDH) activities.

**Objectives:**

Based on these previous studies, we investigated the effects of three natural isoflavones, daidzin, daidzein, and genistein, on the modulation of the cocaine reinforcing effects and on cue-induced reinstatement in an operant mouse model of cocaine self-administration.

**Results:**

Chronic treatment with daidzein or genistein decreased operant responding to obtain cocaine intravenous infusions. On the other hand, daidzein and daidzin, but not genistein, were effective in decreasing cue-induced cocaine reinstatement. Complementary studies revealed that daidzein effects on cocaine reinforcement were mediated through a mechanism that involved dopamine type-2/3 receptors (DA-D2/3) activities.

**Conclusions:**

Our results suggest that these natural compounds alone or in combination can be a potential therapeutic approach for cocaine addiction. Further clinical studies are required in order to ascertain their potential therapeutic use.

**Supplementary Information:**

The online version contains supplementary material available at 10.1007/s00213-021-05820-z.

## INTRODUCTION

Cocaine addiction is a chronic relapsing disorder characterized by compulsive drug seeking and use. Cocaine inhibits the monoamine transporters producing an increase in the concentration of dopamine (DA) and other monoamines in the synaptic cleft in different brain areas, including the mesocorticolimbic pathway, a brain circuit crucially involved in drugs reinforcing effects (Camí and Farré 2003). These pharmacological actions are considered to mediate the reinforcing effects of cocaine and also to be involved in the development of cocaine addiction (Dackis and O'Brien 2001). At present time, no effective treatment for this disorder is available.

Isoflavones are biologically active polyphenols present in soybeans and other plants such as Kudzu (*Pueraria lobata*). Daidzin, daidzein, and genistein, among others, are members of this family of compounds. Studies performed in the lasts decades have described important effects of these isoflavones regulating a variety of neurophysiological responses, including the activity of the mesocorticolimbic dopaminergic system. In this regard, Kudzu root extracts have been used in traditional Chinese medicine for centuries to treat alcoholism (Keung and Vallee 1994). Further research in rodents demonstrated an important role of daidzin in these responses, reducing alcohol consumption by selectively inhibiting the activity of the aldehyde dehydrogenases (ALDH), a family of enzymes involved in dopamine (DA) and ethanol metabolism (Keung et al. 1997). More recent investigations with a synthetic derivative of daidzin called CVT-10216 or GS 455534 also described important inhibitory effects of this compound on alcohol and cocaine consumption, as well as in palatable food binge eating behavior. Changes in ALDH activities and in DA neurotransmission in the mesocorticolimbic system were considered the main neurobiological mechanism involved in the responses observed after CVT-10216 administration (Arolfo et al. 2009; Lange and Diamond 2009; Yao et al. 2010; Bocarsly et al. 2014).

Daidzein, another isoflavone present in Kudzu roots, also produces potent antidipsotropic effects for ethanol in rodents (Keung and Vallee 1993) although, in contrast with daidzin, the mechanism of action does not involve the inhibition of ALDH activities (Keung et al. 1997) and remains to be clarified. Further investigations, however, have described modulatory effects of this isoflavone on DA synthesis and release (Bare et al. 1995) that could account for the inhibitory effects of daidzein on alcohol consumption. On the other hand, genistein also regulates DA synthesis and release, as was described in mouse striatal slices (Goldstein et al. 2012). Moreover, both, genistein and daidzein, show potent agonistic effects on estrogen receptors (ER) activities (Kuiper et al. 1998). The estrogen receptors are known to exert important regulatory effects on dopamine neurotransmission (Lammers et al. 1999) and on cocaine addiction (Bagley et al. 2019).

These earlier investigations suggest that, by a direct or indirect mechanism, isoflavones can regulate DA neurotransmission in the brain reward system and subsequently could be of potential interest for the treatment of cocaine addiction. The aim of this study was to evaluate the effects of daidzin, daidzein and genistein in cocaine reinforcing effects and cue-induced seeking behavior using an operant mouse model of cocaine self-administration. We have also performed complementary studies to elucidate the possible mechanisms of action of these isoflavones on the modulation of cocaine reinforcement. Results obtained were compared with those produced by a reference ALDH inhibitor, disulfiram.

## METHODS

### Animals

Male CD-1 mice from 20 to 22 g (around 28-day old) at the beginning of the experiments were used. Mice were maintained in a temperature (21 ± 1°C) and humidity (65 ± 10%) controlled room with reversed light-dark cycle (lights on from 20:00 to 08:00 h) except for the locomotor activity studies where animals were exposed to a normal light dark cycle (lights on from 8:00 to 20:00 h). Animals trained in the operant self-administrations boxes were single-housed after intravenous catheter surgery. Mice exposed to the locomotor activity studies were housed 4 per cage. Different sets of mice were used on each study of locomotion and self-administration. Behavioral tests and animal care were conducted in accordance with the standard ethical guidelines (European Commission Directive 86/609 EC) and approved by the local ethical committee (Comité Etico de Experimentación Animal, PRBB-UPF). All behavioral studies were performed in blind conditions. All mice were handled and habituated to the conditions of the animal facility for at least 1 week before the experiments started.

### Drugs

Cocaine hydrochloride was obtained from the Spanish Agency of Medicines and Medical Devices (AEMPS, Madrid, Spain), dissolved in sterile saline, and administered at 0.5 mg/kg (intravenously, i.v.). All natural isoflavones (daidzin, daidzein, and genistein) were purchased from LC Laboratories (Woburn, MA) or PhytoLab (Vestenbergsgreuth, Germany), dissolved in 50 μl of Tween 80/0.5% carboxymethylcellulose (CMC) in sterile saline and administered at different doses by intraperitoneal (i.p.) route. Disulfiram was purchased from TOCRIS (Bio-Techne, Minneapolis, MN), dissolved in 50 μl of Tween 80/0.5% CMC in sterile saline and administered at different doses by i.p. route. Phenylephrine (1 mg/kg, subcutaneous (s.c.), not permeable to cross the Blood Brain Barrier), atipamezole (0.4 mg/kg, s.c.), isoproterenol (0.25 mg/kg, i.p.), SKF38393 (0.1 mg/kg, s.c.), quinpirole (0.01 mg/kg, i.p.) and tamoxifen (1 mg/kg, i.p.) were purchased from TOCRIS and dissolved in 50 μl of Tween 80/0.5% CMC in sterile saline.

### Locomotor activity

Locomotion was evaluated in locomotor activity boxes (9 × 20 × 11 cm; Imetronic, France) containing a line of photocells 2 cm above the floor to measure horizontal movements, and another line located 6 cm above the floor to measure vertical activity (rearing). Mice were individually placed in the boxes and the total activity was recorded during 60 min in a low luminosity environment (20–25 lux). Total locomotor activity was evaluated as the sum of the horizontal and vertical movements. All the locomotor activity tests were performed between 09:30 and 16:30 h. Isoflavones and disulfiram were administered 60 min before locomotor activity measurements. Phenylephrine, atipamezole, isoproterenol, SKF38393, quinpirole, and tamoxifen were administered 30 min before locomotor activity was evaluated. On each locomotion experiment, a control group of mice (vehicle) was included. Mice from all experimental conditions were evaluated in a counterbalanced manner. All locomotor activity studies were performed with no previous habituation to the activity boxes.

### Cocaine self-administration procedure

#### Apparatus

Self-administration training and testing occurred in operant chambers (Model ENV-307A-CT, Med-Associates, Georgia, VT, USA) equipped with two holes, one selected as the active hole for delivering the reinforcer and the other as the inactive hole. Acquisition of drug self-administration was performed using a fixed ratio 1 (FR1) schedule of reinforcement such that one nose-poke in the active hole resulted in one cocaine infusion, while nose-poking in the inactive hole had no programmed consequences. A stimulus light, located above the active hole, was paired contingently with the delivery of the reinforcer. Pump noise and a stimulus-light located above active hole were paired with the delivery of the infusion. When mice responded on the reinforced hole, the stimulus light went on, and a cocaine infusion was delivered. Each response on the active manipulandum in this phase led to the presentation of the cue light for 2 s. Infusions were delivered in a volume of 23.5 μl over 2 s. Cocaine was infused via a syringe that was mounted on a microinfusion pump (PHM-100A, Med-Associates, Georgia, VT, USA) and connected, via Tygon tubing (0.96 mm o.d., Portex Fine Bore Polythene Tubing, Portex Limited, Kent, England) to a single channel liquid swivel (375/25, Instech Laboratories, Plymouth Meeting, PA, USA), and to the mouse i.v. catheters. The swivel was mounted on a counter-balanced arm above the operant chamber.

#### Surgery

Mice were anesthetized with a ketamine/xylazine mixture (0.1 ml/10 g body weight, i.p.) and then implanted with indwelling i.v. silastic catheters in the right jugular vein, as previously described (Trigo et al. 2006). After surgery, animals were individually housed and allowed 4 days for recovery before starting the operant training. The patency of the catheters was evaluated at the end of the operant cocaine self-administration experimental sequence, and whenever drug self-administration behavior appeared to deviate more than 50% from the one previously observed, by infusing 0.1 ml of thiopental (5 mg/ml) through the catheter. If prominent signs of anesthesia were not apparent within 3 s of the infusion, the animal was removed from the experiment.

#### Procedure

Animals were trained to nose-poke under an FR1 schedule of reinforcement to receive cocaine (0.5 mg/kg/infusion). Self-administration session started with a priming infusion of the drug, lasted for 60 min and was conducted 7 days a week. After each session, mice were returned to their home-cages. The number of reinforcers was limited to 50 infusions per session. Each infusion was followed by a 15-s time-out period during which an active nose-poke had no consequence. Stable acquisition of self-administration behavior was achieved when the three following conditions were met: (i) <20% deviation from the mean of the total number of reinforcers earned in three consecutive sessions (80% stability), (ii) at least 75% responding on the active hole, and (iii) a minimum of four reinforcers earned per session. When stability criteria were achieved, the effects of daidzin (75 mg/kg, i.p.), daidzein (100 mg/kg, i.p.), genistein (100 mg/kg, i.p.), disulfiram (75 mg/kg, i.p.), and vehicle were evaluated on cocaine self-administration for 5 consecutive days. These compounds were administered 60 min before starting the self-administration session on each day. In a different set of mice, quinpirole or tamoxifen were injected 30 min after vehicle or daidzein (100 mg/kg, i.p.) administration during 5 consecutive days to evaluate the involvement of dopaminergic or estrogen receptors on the modulatory effects of this isoflavone on cocaine self-administration.

Data from acquisition of cocaine self-administration (Fig 2) were expressed as number of infusions and as area under the curve (AUC). AUC was calculated by using a standard trapezoidal method. The following equation was used: AUC = [0.5 *(B1 + B2)* h] + [0.5 *(B2 + B3)* h] +….+ [0.5 *(Bn + Bn + 1)* h], where Bn were the infusions received for each mouse and h was the time (days) passed between the consecutive measurements (Gibaldi and Perrier 1975).

A different set of mice were trained to self-administer cocaine. After reaching the acquisition criteria, mice underwent to an extinction process. During this phase, nose-poking into the reinforced poke caused no consequences, meaning that the pump was turn off and the mice did not receive the infusion of the drug nor the conditioned stimulus (light). Mice were exposed to 1 h daily extinction sessions 6 days a week until extinction criterion was reached. The extinction criterion was achieved when mice made a mean number of active responses in three consecutive extinction sessions of less than 30% of the responses performed during the last day of the cocaine-training period. A small percentage of animals never reached the extinction criteria after the exposure to the extinction protocol for a month and a half (less than around 10%) and were excluded from the experiment.

After achieving the extinction criterion, mice were exposed to cue (stimulus light)-induced reinstatement of cocaine seeking behavior. Cue-induced reinstatement was conducted under the same conditions used in the acquisition phase except that cocaine was not delivered. Each response on the active manipulandum in this phase led to the presentation of the cue light for 2 s and the activation of the pump (but with no delivery of the drug). Animals were reexposed to several sessions of cue-induced reinstatement using a Latin-square design. Each reinstatement session was preceded by an extinction period and not performed until mice reached the extinction criterion again. This methodological approach allowed evaluating in the same animal the effects of vehicle and one isoflavone or disulfiram on cue-induced relapse. The doses used and period of administration of these compounds were the same as when tested during cocaine self-administration (75 or 100 mg/kg, i.p., 60 min before testing), but the animals received only an acute injection. We also followed a Latin-square design to investigate the involvement of the dopaminergic (quinpirole) or estrogen (tamoxifen) receptors modulating daidzein or daidzin effects on cue-induced cocaine relapse. These compounds were injected 30 min after vehicle or isoflavone administration.

All mice used on cocaine self-administration studies and on cue-induced relapse studies were distributed in the different experimental groups homogeneously based on their responses during the cocaine self-administration period.

### Statistical analysis

Locomotor activity results were analyzed for each compound evaluated by using one-way ANOVA followed by Dunnett's multiple comparison tests when required. The involvement of the estrogen, dopaminergic, and adrenergic systems on daidzein-induced hypolocomotor effects were studied employing two-way ANOVA models including condition (vehicle vs. daidzein) and treatment (vehicle, phenylephrine, atipamezole, isoproterenol, SKF38393, tamoxifen, quinpirole) as well the interaction of both factors. All post hoc pairwise comparisons of treatment versus vehicle under both conditions were carried out in the framework of that model. The interaction permitted to compare each treatment versus the vehicle separately for both conditions by means of post-hoc comparisons.

We analyzed the effects of the isoflavones or disulfiram on active responding during cocaine self-administration by means of four two-way repeated measures ANOVA models including day (of treatment) and treatment (daidzein, daidzin, genistein, or disulfram versus vehicle) as well as their interaction as factors. In case the day-treatment interaction was statistically significant, treatment comparisons were carried out separately for each day, otherwise no post-hoc comparisons were performed. The area under the curve (AUC) of the active responding was also calculated for each compound in the acquisition of cocaine self-administration and analyzed with one-way repeated measures ANOVA model.

Tamoxifen and quinpirole modulatory effects on active cocaine self-administration were evaluated separately for control vehicle- and daidzein-treated mice by using 2-way repeated measures ANOVA models that included day (of treatment) and treatment (vehicle, tamoxifen, quinpirole) as well as the interaction of all both factors. Data shown on Figs. 2 and 3 and the corresponding statistical analysis was performed only with the number of active nose-poke responses when cocaine was available. Active nose-pokes during the time out period were not included.

The model assumptions of all models, that is, homoscedasticity and normality, were checked by means of different residual plots. Whenever the model assumption did not seem to hold, the data were log-transformed prior to the analyses. All post-hoc pairwise comparisons were carried out in the framework of the corresponding model and the computation of the simultaneous confidence intervals and adjusted *p* values in order to guarantee a family-wise error rate of 0.05 was based on the multivariate t distribution of the vector of test statistics (Hothorn et al. 2008).

Isoflavone or disulfiram effects on the active nose-poke responses after cue-induced cocaine reinstatement were analyzed by applying a paired Student's *t*-test analysis.

Finally, the effects of quinpirole and tamoxifen modulating the active nose-poke responses on cue-induced cocaine reinstatement were analyzed separately for vehicle- or daidzein- or daidzin-treated animals by applying a one-way ANOVA, followed by Dunnett's post hoc analysis tests when required.

All data were analyzed using the statistical software R, version 3.4.3. (Vienna, Austria; http://www.rproject.org) or STATISTICA software. Results were considered statistically significant whenever *p* < 0.05. All the results are expressed as mean + SEM.

## RESULTS

### Daidzin, daidzein, genistein, and disulfiram effects on locomotor activity in CD-1 male mice

This preliminary experiment was carried out in order to select the appropriate dose of each isoflavone that produce no locomotor effects to be used later on in the cocaine self-administration studies. The range of doses of each isoflavone and disulfiram tested in this experiment was selected based on previous studies (Keung and Vallee 1993; Schroeder et al. 2010). Daidzein and genistein were administered at the doses of 100, 200, and 400 mg/kg, i.p.; whereas daidzin and disulfiram at the doses of 75, 150, and 300 mg/kg, i.p.. Mice were exposed to the locomotor activity boxes 60 min after the administration of the corresponding compound.

**Fig. 1:**
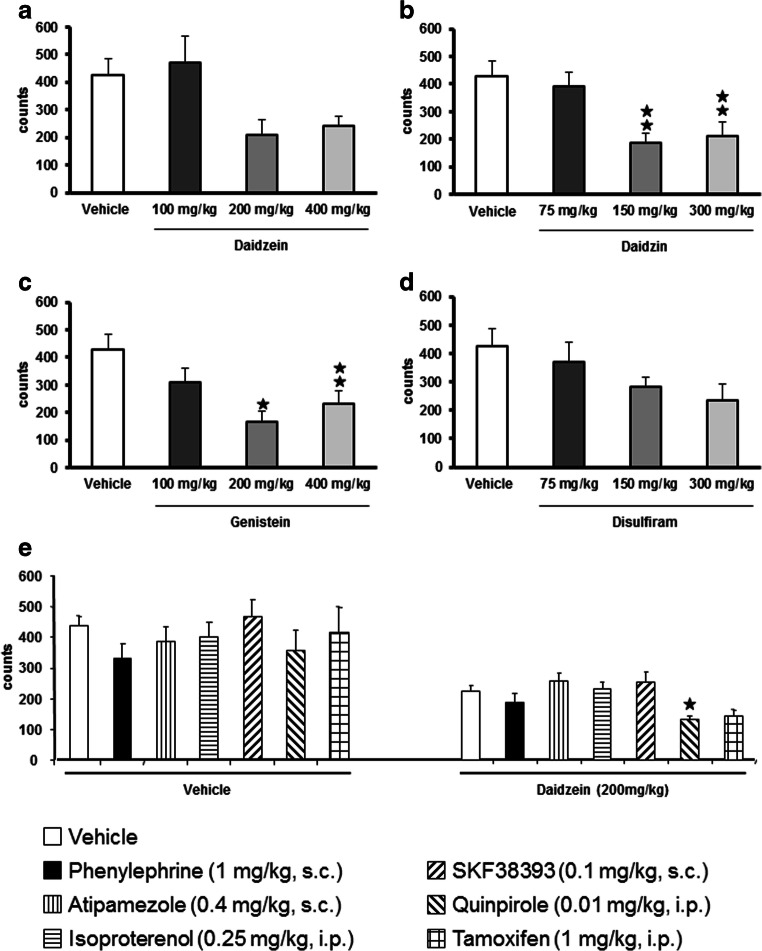
Locomotor effects induced after acute isoflavone or disulfiram administration. Involvement of dopamine D2/3 and estrogen-receptor systems in the locomotor effects induced by daidzein. Total locomotor activity 60 min after acute administration (i.p.) of different doses of (a) daidzein (*n* = 8-10), (b) daidzin (*n* = 8-10), (c) genistein (*n* = 8-10), or (d) disulfiram (*n* = 8-10). Effects of acute administration (30 min before locomotion was evaluated) of different agonist or antagonist for the adrenergic, dopaminergic or estrogen receptor systems (*n* = 9-15 per group) in the locomotor activity induced by (e) acute vehicle (60 min before locomotion) or daidzein (200 mg/kg, 60 min before locomotion) administration. A total of 9-15 mice were included per experimental group. Results are expressed as average + SEM, ⋆ ⋆ *p* < 0.01, ⋆ *p* < 0.05 versus vehicle or versus daidzein-vehicle treated mice, post hoc pairwise comparisons

Results show that only daidzin and genistein produced some hypolocomotor effects when administered at the highest doses (*F*_(3,30)_ = 6.7093, *p* < 0.001; *p* < 0.01 vehicle vs. daidzin 150 and 300 mg/kg) (Fig. 1b); (*F*_(3,25)_ = 4.809, *p* < 0.01; *p* < 0.01 vehicle vs. genistein 200 mg/kg and *p* < 0.05 400 mg/kg) (Fig. 1c). Daidzein and disulfiram did not produce substantial locomotor changes at any of the doses tested, although the highest ones tended to reduce locomotor activity (*F*_(3,30)_ = 3.8821, n.s and *F*_(3,28)_ = 2,369, n.s, respectively) (Fig. 1a,d). Based on these results, daidzein and genistein at the dose of 100 mg/kg, i.p., 60 min, and daidzin and disulfiram at the dose of 75 mg/kg, i.p., were chosen to be evaluated in the operant cocaine self-administration paradigm.

### Daidzein produces its hypolocomotor effects through a mechanism that requires dopaminergic receptor activities in CD-1 male mice

Another preliminary locomotor study was conducted to delve into the mechanism regulating isoflavone pharmacological actions. We investigated the involvement of the estrogen, noradrenergic and dopaminergic systems in these responses. Because preliminary studies in the laboratory suggested that daidzein effects modulating cocaine reinforcing were more relevant than those produced by daidzin or genistein, this study was conducted in animals treated with daidzein.

Animals were exposed to vehicle or to daidzein (200 mg/kg, i.p., a dose known to produce hypolocomotor effects). Thirty minutes later animals received a second administration of vehicle or a subeffective dose of one of the following compounds: tamoxifen (an estrogen receptor antagonist, 1 mg/kg, i.p.), phenylephrine (a peripheral α-1 adrenergic receptor agonist, 1 mg/kg, s.c), atipamezole (an α-2 adrenergic receptor antagonist, 0.4 mg/kg, s.c.), isoproterenol (a β-adrenergic agonist, 0.25 mg/kg, i.p.), SKF38393 (a DA type-I, D1, receptor agonist, 0.1 mg/kg, s.c.), or quinpirole (a dopamine type-II/III, D2/3, receptor agonist, 0.01 mg/kg, i.p.). Thirty minutes after the second administration locomotion was evaluated for a period of 60 min (Fig. 1e).

The statistical analysis revealed that the doses chose of each estrogen, adrenergic, or dopaminergic compounds were subeffective, as no significant differences were observed in between the different groups of mice treated with vehicle (Fig. 1e). Daidzein (200 mg/kg, i.p.) produced significant hypolocomotor effects when compared with control vehicle treated mice (*p* < 0.05).

Two-way ANOVA also demonstrated a significant effect of the treatment with daidzein (*F*_(1,129)_ = 66.130, *p* < 0.001), with an effect of the posterior treatment with the different agonists or antagonists (*F*_(6,129)_ = 4.195, *p* < 0.001) with no interaction between both factors (*F*_(6,129)_ = 1.324, n.s.).

Further statistical analysis also revealed that the treatment with a subeffective dose of the D2/3 dopaminergic receptor agonist quinpirole (0.01 mg/kg) (Fig. 1e) modified daidzein-induced hypolocomotion (*p* < 0.05 post hoc pairwise comparisons: daidzein-vehicle vs. daidzein-quinpirole). Tamoxifen treatment (1 mg/kg, i.p.) also modified daidzein hypolocomotion, although it did not reach statistical significance (*p* = 0.084).

Daidzein, like other isoflavones, can produce blood vessel relaxation that may alter blood pressure (Nevala et al. 2001) and indirectly affect locomotion. In order to discard the involvement of hypotensive changes on daidzein hypolocomotor effects, the combination with phenylephrine, a peripheral α-1 adrenergic receptor agonist with well-known hypertensive properties, was also evaluated. No statistically significant differences in locomotion were observed after phenylephrine administration (Fig. 1e), suggesting that daidzein hypolocomotor responses were not the consequence of associated alterations in blood pressure.

Moreover, any of the others agonists or antagonists tested in this locomotor study (atipamezole, isoproterenol or SKF38393, Fig. 1e) did not produce statistically significant effects when evaluated.

### Chronic treatment with daidzein decreases cocaine reinforcement in CD-1 male mice

In order to evaluate the effects of daidzein, daidzin, genistein, and disulfiram on cocaine reinforcement, a new set of mice underwent surgery and got implanted with i.v. catheters and trained in operant boxes to actively nose-poke to obtain infusions of cocaine (0.5 mg/kg/infusion). After reaching the stability criteria, animals were divided into the different experimental groups and received vehicle, daidzein (100 mg/kg, i.p.), daidzin (75 mg/kg, i.p.), genistein (100 mg/kg, i.p.), or disulfiram (75 mg/kg, i.p.) for 5 consecutive days 60 min before being exposed to the self-administration task.

**Fig. 2:**
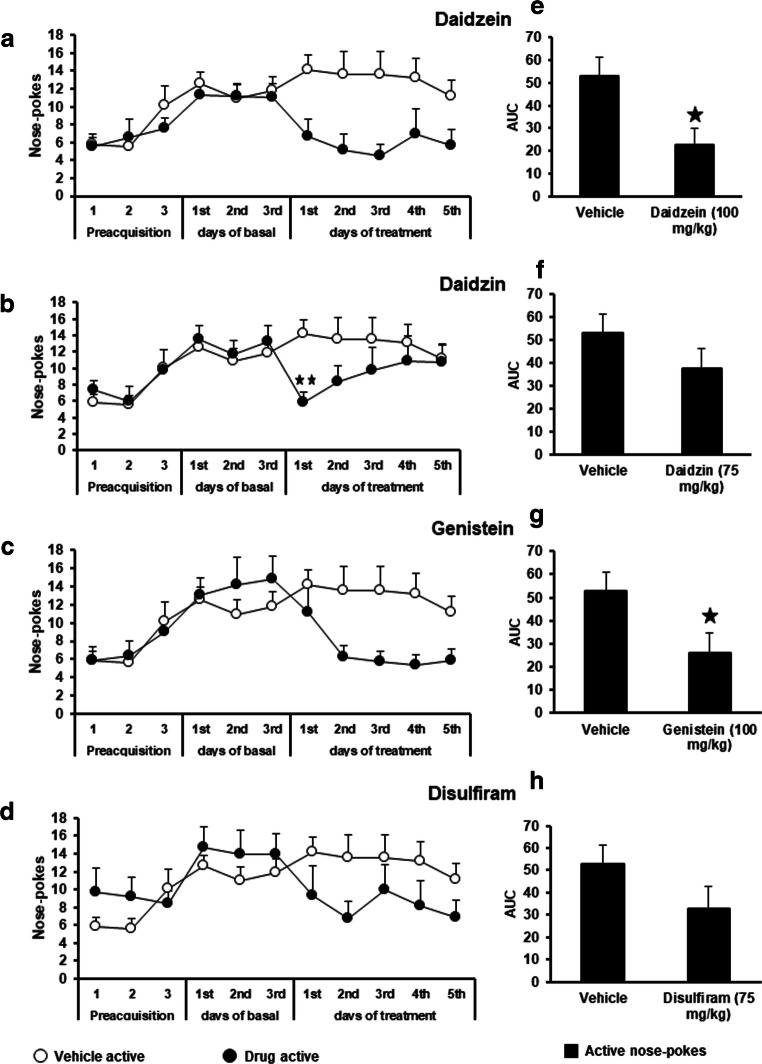
Chronic treatment with isoflavones decreases operant responding for cocaine. (a–d) Effects of the chronic treatment with vehicle (*n* = 12) and daidzein (100 mg/kg/day, i.p., *n* = 11) (a), daidzin (75 mg/kg/day, i.p., *n* = 10) (b), genistein (100 mg/kg/day, i.p., *n* = 10) (c) and disulfiram (75 mg/kg/day, i.p., *n* = 9) (d) on cocaine self-administration. (e–h) Area under the curve of the 5 days of chronic treatment with vehicle (*n* = 12) and daidzein (100 mg/kg/day, i.p., *n* = 11) (e), daidzin (75 mg/kg/day, i.p., *n* = 10) (f), genistein (100 mg/kg/day, i.p., *n* = 10) (g) and disulfiram (75 mg/kg/day, i.p., *n* = 9) (h) on cocaine self-administration. Animals received the injection of the vehicle, isoflavone or disulfiram for 5 consecutive days 1 h before the beginning of the operant training session. Data are represented as the mean number of nose-pokes in the active hole to obtain cocaine (0.5 mg/kg/infusion) during the preacquisition and acquisition-training, and following the chronic treatment for 5 days with vehicle, isoflavone or disulfiram. Data are expressed as mean + SEM. ⋆ ⋆ *p* < 0.01, ⋆ *p* < 0.05 versus vehicle treated mice post hoc pairwise comparisons

When the effects of chronic daidzein (100 mg/kg, i.p.) versus chronic vehicle treatment were compared in the active nose-poke responding, two-way repeated measures ANOVA revealed no day-treatment interaction (*F*_(4,84)_ = 0.862, n.s.). For this reason, the interaction was removed from the model and the updated model revealed a treatment effect (*F*_(1,21)_ = 17.744, *p* < 0.001), but no effect of the day (*F*_(4,88)_ = 0.991, n.s.). The estimated treatment difference (on the log-scale) of daidzein versus chronic vehicle was −1.169 (95% CI: [−1.712, −0.625]; Fig. 2a).

Chronic daidzin (75 mg/kg, i.p.) effects on active nose-poke responding were also analyzed and compared with vehicle-treatment. Two-way ANOVA of repeated measures demonstrated no effect of the days (*F*_(4,80)_ = 0.410, n.s.), an effect of the treatment (*F*_(1 20)_ = 4.538, *p* < 0.05) and an interaction between both factors (*F*_(4,80)_ = 3.687, *p* < 0.01). Further analysis demonstrated a significant difference in the active responding in between mice treated with vehicle and daidzin on day 1 (Fig. 2b; day 1 (*p* < 0.01)) but not after the chronic exposure to this isoflavone.

When the effects of chronic genistein (100 mg/kg, i.p.) versus chronic vehicle treatment were compared in the active nose-poke responding, two-way ANOVA of repeated measures revealed no day-treatment interaction (*F*_(4,80)_ = 0.513, n.s.). For this reason, the interaction was removed from the model and the updated model revealed a treatment effect (*F*_(1,20)_ = 14.969, *p* < 0.001), but no effect of the day (*F*_(4,84)_ = 1.557, n.s.). The estimated treatment difference (on the log-scale) of genistein versus chronic vehicle was −0.786 (95% CI: [−1.184, −0.3878]; Fig. 2c).

Statistical analysis (two-way ANOVA repeated measures) revealed no significant day-treatment interaction (*F*_(4,76)_ = 1.051, n.s.) in the active responding of mice treated with disulfiram when compared with the animals treated with vehicle. Update analysis (after removing the interaction) revealed a treatment effect (*F*_(1,19)_ = 6.648, *p* < 0.05), but no effect of the day (*F*_(4,80)_ = 2.009, n.s.). The estimated treatment difference (on the log-scale) of disulfiram versus chronic vehicle was −0.760 (95% CI: [−1.338, −0.182]; Fig. 2d).

The AUC of active cocaine self-administration responses was also calculated for each compound and compared with the control vehicle treated animals. Results obtained demonstrated that daidzein significantly decreased cocaine self-administration responses (*p* < 0.05 versus vehicle treated animals; Fig. 2e). Interestingly, the statistical analysis also revealed a significant effect of genistein modifying cocaine self-administration responses (*p* < 0.05 vs. vehicle; Fig. 2g). Modifications in the AUC after daidzin and disulfiram treatment were also calculated. No significant effects in the one-way ANOVA were revealed after daidzin or disulfiram administration (n.s. in both cases; Fig. 2f,h).

On the other hand, responses in the inactive nose-poke were not modified by any treatment, displaying an average response ranging from 1.7 to 2.8 in between the different experimental groups (see Suppl. Fig. 1).

These results suggest that isoflavone effects on drug reinforcement are heterogeneous and point to different mechanisms of action involved in the regulatory actions produced by each isoflavone.

### Involvement of the D2/3 dopamine receptor on daidzein-induced modulatory effects in cocaine self-administration in CD-1 male mice

Based on our previous results obtained in the locomotor activity studies (Fig.1), we investigated the involvement of the estrogen receptors (tamoxifen produced clear modulatory actions on daidzin locomotor effects that almost reached statistical significance, see Fig. 1e) and DA D2/3 receptors in the modulatory effects induced by daidzein (isoflavone)s on cocaine reinforcement.

Daidzein was selected as a reference isoflavone for this study because preliminary experiments (included in the results shown in Figs. 2 and 4) demonstrated more relevant effects modulating both, active cocaine self-administration and cue-induced seeking behavior than those produced by daidzin or genistein.

**Fig. 3:**
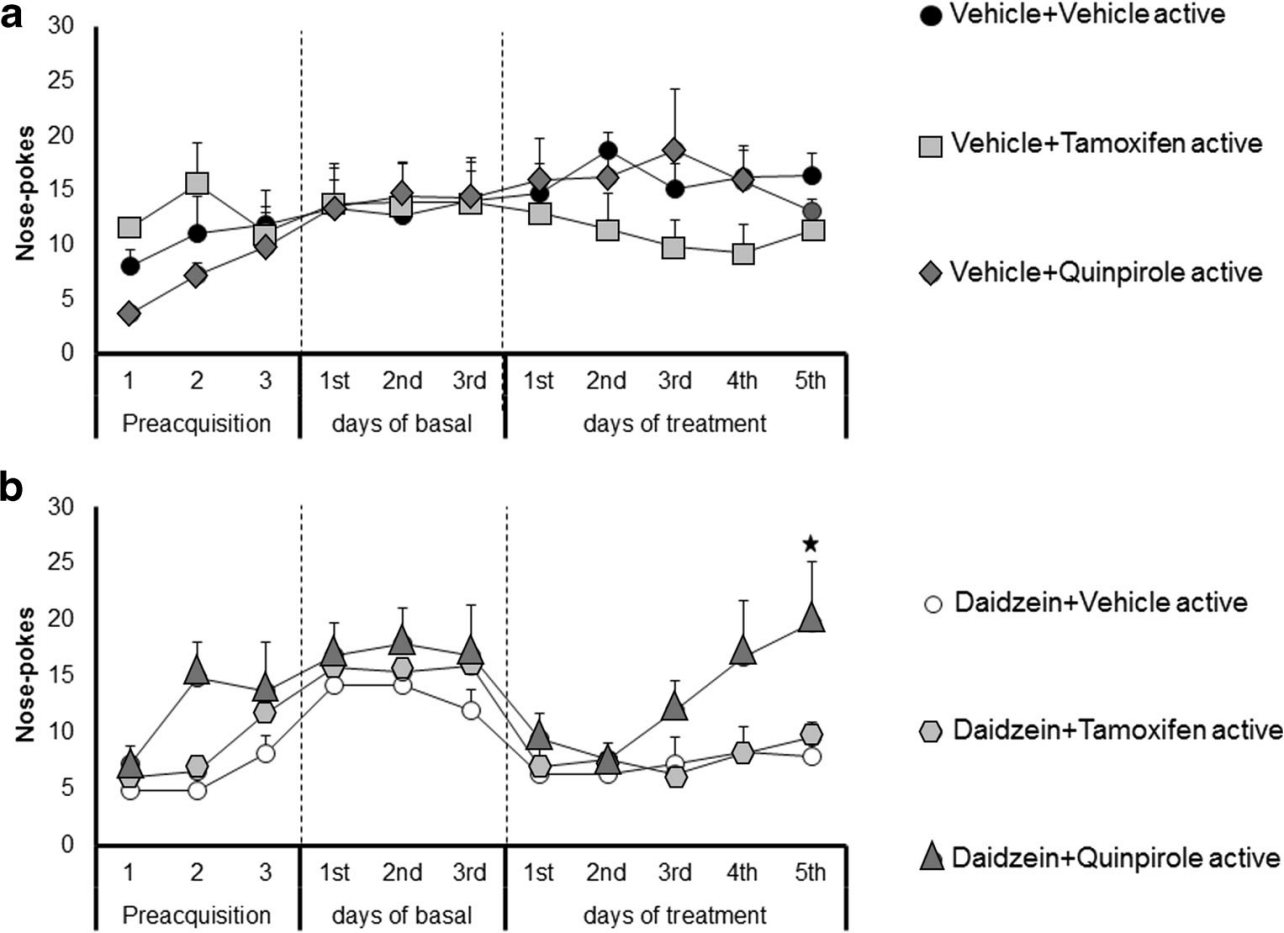
Involvement of the dopamine D2/3 receptor on daidzein-induced modulatory actions in cocaine self-administration paradigm. Effects of the exposure to vehicle, quinpirole (0.01 mg/kg, i.p.) or tamoxifen (1 mg/kg, i.p.) in the operant responding to obtain cocaine in mice previously treated with (a) vehicle (i.p., *n* = 7-10) or (b) daidzein (100 mg/kg/day, i.p., *n* = 8-9). Data are represented as the mean number of nose-pokes in the active hole to obtain cocaine (0.5 mg/kg/infusion) during the preacquisition and acquisition-training, and following the chronic treatment for 5 days with vehicle or daidzein (60 min before the beginning of the training session) and subsequent administration of vehicle, quinpirole, or tamoxifen (30 min before the beginning of the training session). Results are expressed as average + SEM. ⋆ *p* < 0.05 versus daidzein-vehicle treated animals on the same day, post hoc pairwise comparisons

Animals were trained to self-administer cocaine. After reaching stability criteria a chronic treatment for 5 consecutive days began. Tamoxifen (1 mg/kg, i.p.), quinpirole (0.01 mg/kg, i.p.) or vehicle were administered 30 min after daidzein (100 mg/kg, i.p.) or vehicle injections during these 5 consecutive days. Thirty minutes later, animals were exposed to the self-administration task. Chronic exposure to quinpirole (0.01 mg/kg, i.p.) or tamoxifen (1 mg/kg, i.p.) produced no significant effects in control animals preexposed to vehicle (Fig. 3a).

Two-way ANOVA of repeated measures revealed a significant effect of the day (*F*_(4,72)_ = 3.319, *p* < 0.05), with no effect of the treatment (*F*_(2,18)_ = 2.313, n.s.) nor interaction between factors (*F*_(8,72)_ = 1.746, n.s.) in the active nose-poke responses in mice exposed to daidzein (100 mg/kg, i.p.) for 5 days. Further analysis show that chronic tamoxifen administration produced no effect in the operant responding in mice pre-treated with this isoflavone (Fig. 3b). However, post-hoc pairwise comparisons revealed a significant effect on the 5th day of the treatment with quinpirole in the active responses in mice exposed to daidzein (*p* < 0.05, daidzein-quinpirole vs. daidzein-vehicle) (Fig. 3b).

On the other hand, responses in the inactive nose-poke were not modified by any treatment, displaying an average response ranging from 0.7 to 1.8 in between the different experimental groups (see Suppl. Fig. 2).

Our results suggest that daidzein modifies cocaine self-administration behavior by a mechanism that requires DA D2/3 receptor activities.

### Daidzein, daidzin and disulfiram, but not genistein, decreased cue-induced cocaine seeking behavior and relapse in CD-1 male mice

A new set of animals was exposed to cocaine self-administration, followed by an extinction protocol. After reaching extinction criteria, the effects of an acute injection of daidzein (100 mg/kg, i.p.), daidzin (75 mg/kg, i.p.), genistein (100 mg/kg, i.p.), or disulfiram (75 mg/kg, i.p.) were evaluated on cue-induced cocaine seeking behavior by applying a Latin square experimental design. All compounds were administered 60 min before starting the relapse session.

**Fig. 4:**
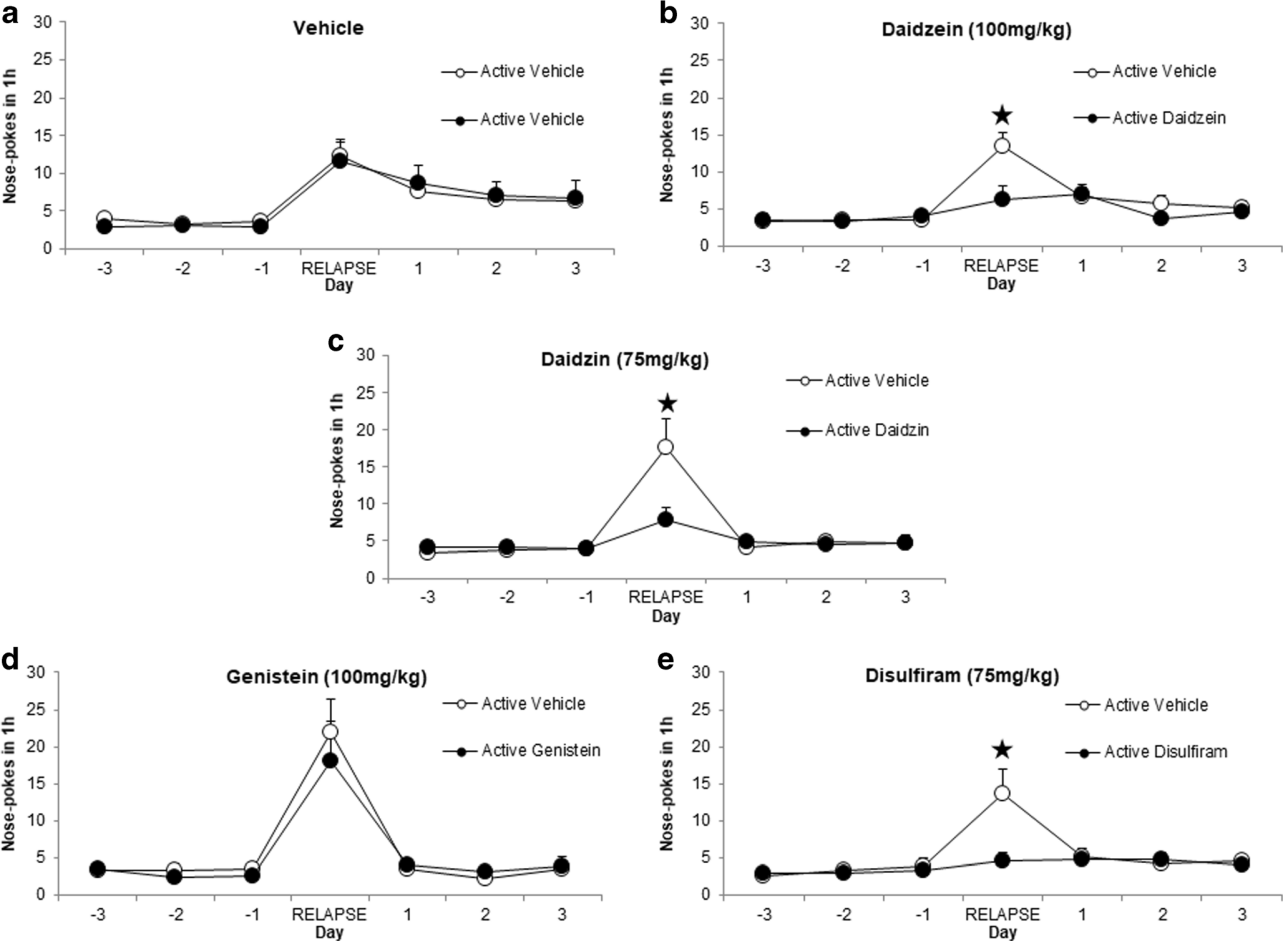
Acute administration of daidzein, daidzin, and disulfiram, but not genistein, decreases cue-induced cocaine seeking and reinstatement behavior. Comparison of the effects of the administration of vehicle versus (a) vehicle (*n* = 9), (b) daidzein (100 mg/kg, i.p., *n* = 9), (c) daidzin (75 mg/kg, i.p., *n* = 9), (d) genistein (100 mg/kg, i.p., *n* = 9) or (e) disulfiram (75 mg/kg, i.p., *n* = 8) in the active nose-poke responding after exposure to cue-induced cocaine seeking behavior paradigm. All compounds were administered acutely 1 h before the beginning of each cue-induced reinstatement session. Data represents the active nose-poke responding the day of exposure to cue-induced reinstatement and also during the extinction period 3 days before and after. Results are expressed as average + SEM. ⋆ *p* < 0.05 versus vehicle-induced reinstatement, Student's *t* test

Paired Student *t*-test analysis revealed that responding in the active hole was not modified in between sessions in mice treated with vehicle (Fig. 4a).

Statistical analysis revealed that daidzein (100 mg/kg, i.p.) decreased cue-induced relapse when compared with vehicle administration (*p* < 0.05) (Fig. 4b). Moreover, daidzin (75 mg/kg, i.p.) preexposure was also effective to decrease cocaine relapse (*p* < 0.05). Similar results were also observed in mice pretreated with disulfiram (75 mg/kg, i.p.), showing a significant decreased in the active nose-pokes responses when compared with vehicle pretreatment (*p* < 0.05) (Fig. 4e).

However, genistein (100 mg/kg, i.p.) did not modify the behavioral responses in this paradigm. Mice treated with this isoflavone showed the same number of active responses as when received vehicle injections (paired Student's *t* test, n.s.) (Fig. 4d).

### Involvement of D2/3 dopamine receptors on daidzein, but not daidzin, modulatory effects in cue-induced cocaine seeking behavior in CD-1 male mice

The involvement of estrogen and DA D2/3 receptors on isoflavone effects modulating cue-induced cocaine seeking behavior were also investigated. The effects of tamoxifen and quinpirole were evaluated in daidzein, and also daidzin treated mice, because of the similar chemical structure shared by both isoflavones.

**Fig. 5:**
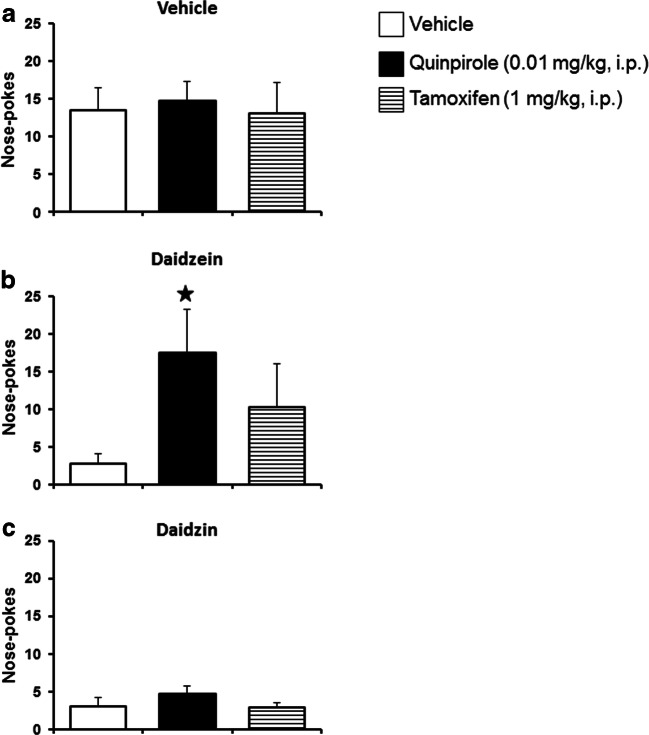
Involvement of the estrogen and dopamine type-II-receptor systems on daidzein-, but not daidzin-, effects in cue-induced cocaine seeking and reinstatement behavior. Comparison of the effects of an acute administration of vehicle or a subeffective dose of quinpirole (0.01 mg/kg, i.p.) or tamoxifen (1 mg/kg, i.p.) on cue-induced cocaine seeking behavior and reinstatement in mice acutely treated with (a) vehicle, (b) daidzein (100 mg/kg, i.p.) or (c) daidzin (75 mg/kg, i.p.). Animals were injected with vehicle, daidzein, or daidzin and 30 min later with tamoxifen, quinpirole, or vehicle. Thirty minutes after the last injection, animals were exposed to cue-induced cocaine seeking behavior paradigm. Results are expressed as average + SEM. ⋆ *p* < 0.05 versus daidzein-vehicle induced reinstatemnet, Dunnett's multiple comparison test

After reaching extinction criteria, animals received vehicle, daidzein or daidzin and 30 min later tamoxifen, quinpirole or vehicle. Thirty minutes after the last injection, animals were exposed to cue-induced relapse. Both tamoxifen and quinpirole did not modify cocaine-seeking behavior in control mice previously treated with vehicle (one-way ANOVA *F*_(2,30)_ = 0.0639, n.s.) (Fig. 5a). Daidzein (100 mg/kg, i.p.) decreased the number of active responses after cue-induced cocaine relapse when compared with vehicle-treated animals (Fig. 5b). One-way ANOVA revealed a significant effect of treatment with quinpirole or tamoxifen (*F*_(2,26)_ = 3.9221, *p* < 0.05). Subsequent post hoc analysis demonstrated that DA D2/3 receptor agonist quinpirole (Fisher's *F* test post hoc analysis, *p* < 0.05), but not estrogen receptor agonist tamoxifen (Fisher's *F* test post hoc analysis, n.s.), significantly modified daidzein effects in the operant responses when compared with vehicle treatment (Fig. 5b).

In addition, acute administration of daidzin also decreased cue-induced cocaine seeking behavior when compared to vehicle treated mice. In contrast with daidzein, administration of tamoxifen or quinpirole did not modify the effects of daidzin on operant responding (*F*_(2,25)_ = 1.0989, n.s.) (Fig. 5c).

These data suggest that daidzein effects on cocaine seeking behavior and relapse are mediated by a mechanism that involves DA D2/3 receptor activities. Moreover, our data suggest that isoflavones, daidzein and daidzin, act through different mechanisms of action regulating cocaine seeking responses.

## DISCUSSION

In the present study, we have demonstrated that the natural isoflavones daidzein, daidzin and genistein can regulate the reinforcing properties of cocaine and also cocaine seeking behavior in an operant self-administration paradigm in mice.

We have compared the effects of these compounds with those produced by disulfiram, a drug known to yield different pharmacological effects including the inhibition of ALDH activities and to reduce alcohol intake (Hald and Jacobsen 1948; Johansson 1992). This compound has recently been evaluated for the treatment of cocaine addiction in humans (Pani et al. 2010) (however, see also Carroll et al. 2016). Our study reveals that daidzein is a promising isoflavone regulating cocaine reinforcing effects (Figs. 2b and 3b) and cocaine relapse (Figs. 4b and 5b). We have also described that daidzein modifies these behavioral responses by a mechanism that requires of DA D2/3 receptor, but not estrogen receptor, activities. This hypothesis was first supported from results obtained in the preliminary locomotor activity study. We observed that exposure to a sub-effective dose of quinpirole potentiated daidzein hypolocomotor effects without affecting the activity of the control vehicle treated mice (Fig. 1e). Furthermore, dopamine D2/3 receptors also modulated daidzein pharmacological actions on both, cocaine self-administration and cocaine induced seeking behavior and relapse. Interestingly, chronic exposure to quinpirole was required to revert the effects of daidzein on cocaine reinforcement (Fig. 3a) whereas an acute administration of quinpirole was sufficient to modify the actions of this isoflavone on cue-induced cocaine seeking behavior and relapse (Fig. 5b). The presence of cocaine in the organism during the active cocaine self-administration and its absence when cue-induced relapse was evaluated could account for the different effectivity of quinpirole (single vs repeated administration) in modulating daidzein actions on both behavioral paradigms. Other mechanisms, however, may also be involved in these responses. In this regard, neurobiological alterations, such as the changes that develop during active cocaine self-administration or after extinction on cue-induced seeking behavior in the dopaminergic and in other neurotransmitters systems in the brain reward system may play an important role. These modifications seem to be characteristic and exclusive of each phase of the addictive process (Samuvel et al. 2008; Wydra et al. 2015; Siciliano et al. 2016) and can also differentially influence the pharmacological effects of the administration of daidzein alone or in combination with quinpirole. However, indirect actions through other neuromodulators/neurotransmitter systems known to modify DA neurotransmission that can also be modulated by daidzein administrations, such as CRF or CCK (Crawley 1991; Wanat et al. 2013; Ahmed et al. 2017), may also encompass these effects. Our observations suggest that daidzein is a natural isoflavone with a therapeutic potential to treat or palliate cocaine reinforcing effects. Its pharmacological mechanism seems to involve alterations in the dopaminergic neurotransmission; however, future investigations are required in order to further characterize it.

In sharp contrast with daidzein, disulfiram exposure produced small effects on cocaine self-administration (Fig. 2d,h). These results are similar to previous preclinical studies in rats describing no effect of an acute disulfiram administration in the operant cocaine self-administration paradigm (Schroeder et al. 2010). When administered in vivo, disulfiram is metabolized into diethylthiomethylcarbamate and other compounds with different pharmacological profiles. The lack of effects of disulfiram could be a reflection of the wide spectrum of pharmacological actions produced by this compound and its metabolites, some of which may show antagonistic effects in the modulation of cocaine reinforcing effects. On the other hand, our results demonstrate that responses in the cue-induced relapse paradigm were decreased after disulfiram treatment (Fig. 4e). These observations are also in agreement with previous preclinical observations describing the inhibitory properties of this compound on cocaine craving and relapse (Suh et al. 2006; Schroeder et al. 2010). Different mechanisms of action have been suggested to explain these effects. Thus, previous investigations have proposed that disulfiram can decrease cue-induced cocaine seeking behavior by altering DA release after the inhibition of ALDH activities. However, other authors suggest that disulfiram can regulate cue-induced cocaine relapse by a different mechanism that involves inhibitory effects on dopamine β-hydroxylase (DBH) activities, the enzyme responsible for the conversion of DA to noradrenaline (NE) in the final step of NE biosynthesis (Weinshenker and Schroeder 2007; Gaval-Cruz and Weinshenker 2009). By decreasing NE release, these authors suggest that disulfiram also reduces mesolimbic dopaminergic tone and cocaine seeking behavior (Gaval-Cruz and Weinshenker 2009). Others, however, propose the opposite that disulfiram ability to reduce cocaine relapse is due to an increase in the dopaminergic tone after DHB inhibition (Bourdélat-Parks et al. 2005; Sofuoglu and Kosten 2006; Devoto et al. 2012). Further research is required in order to ascertain the correct mechanism of action involved in these responses.

Acute administration of daidzin decreased operant cocaine self-administration (Fig. 2b). However, tolerance to its pharmacological effects developed when mice were exposed to after a more prolonged treatment (Fig. 2b). Furthermore, the acute administration of daidzin also decreased cocaine seeking behavior in the cue-induced relapse paradigm (Fig. 4c). Previous studies performed with this isoflavone and with some of its synthetic analogues, such as CVT-10216, suggest that daidzin can decrease cocaine effects by a mechanism that involves modulatory actions in ALDH-2 and DA activities (Keung et al. 1997; Arolfo et al. 2009; Yao et al. 2010). Our results, however, suggest that DA D2/3 receptors may play a marginal role in modulating daidzin pharmacological effect. This statement is based on our observations in the cue-induced relapse paradigm, where no differences were observed after exposure to quinpirole in daidzin treated mice (Fig. 5b). We can suggest that other dopamine receptor, like type-I receptors, may play a more prominent role in daidzin pharmacological effects. Moreover, the regulation of ALDH-2 by this isoflavone can also lead to alterations in 5-HT metabolism (Keung and Vallee 1998) that can influence cocaine addictive properties. In this sense, previous studies have shown that 5-HT modifies cocaine reinforcement, seeking behavior and relapse by a mechanism that requires interactions with DA and/or glutamate neurotransmission (Howell and Cunningham 2015).

Genistein was also effective decreasing cocaine reinforcing effects (Fig. 2c,g), an effect that developed after the second day of exposure to this isoflavone. However, in sharp contrast with daidzein and daidzin, genistein did not modify the active nose-poke response in the cue-induced cocaine relapse paradigm (Fig. 4d). Different neurobiological mechanism may be involved in the regulatory effects of genistein modulating cocaine reinforcing effects. In this regard, genistein is a potent agonist of the β-estrogen receptors (Wang et al. 1996), and previous studies have shown the involvement of these receptors modulating cocaine-rewarding effects by a mechanism not fully understood. Thus, blocking the brain estrogen receptors activity completely inhibits the development and expression of cocaine-induced locomotor sensitization and cocaine reinforcing properties in rodents (Segarra et al. 2014; Satta et al. 2018). On the other hand, genistein is also a potent inhibitor of tyrosine kinase (TK) activities and previous studies have shown that genistein can increase DA release from mouse striatal slices by modifying the activity of this enzyme (Bare et al. 1995). Moreover, genistein can also modulate different intracellular signaling pathways, including the nuclear factor-kappa β (NF-kβ) (Kazi et al. 2003; Nagaraju et al. 2013) which is known to be increased in the nucleus accumbens after cocaine administration. This increase in neuronal NF-kβ signaling pathways has been implicated in the long-term effects of cocaine that lead to the development of cocaine addiction (Ang et al. 2001). Thus, different mechanisms might play a role in the effects produced by genistein on cocaine reinforcing effects, however, further research is required in order to ascertain and deeply understand the complexity of these responses. A clinical study with these compounds, especially daidzein, is required to ascertain their promising therapeutic use for the treatment of cocaine addiction in humans. Moreover, the use of natural compounds may be safer and promising for human purposes (Munro et al. 2003; Choi and Rhee 2006;); although caution should be taken into consideration if administered at higher doses like in this study. Each isoflavone produces its effects on cocaine addiction modulating different neurotransmitter, receptor systems and intracellular pathways so further research is required in order to unravel each specific mechanism. Moreover, under a clinical therapeutic perspective, it will be important to take into consideration whether treatment to patients should be performed with soybean extracts or with specific isoflavones.

In summary, there is a need for further studies on the mechanisms of plant extracts and their active compounds action, like isoflavones, that might be a valuable alternative way for the prevention and treatment of various drug dependences.

## Supplementary Information

Supplementary Figure 1: Inactive nose-poke responses in mice treated with isoflavones during operant responding for cocaine. (A to D) Effects of the chronic treatment with vehicle (n=12) and daidzein (100 mg/kg/day, i.p., n=11) (A), daidzin (75 mg/kg/day, i.p., n=10) (B), genistein (100 mg/kg/day, i.p., n=10) (C) and disulfiram (75 mg/kg/day, i.p., n=9) (D) on the inactive nose-poke responding during cocaine self-administration. Results are expressed as average + SEM (PNG 550 kb)

High resolution image (TIF 85 kb)

Supplementary Figure 2: Inactive nose-poke responses in mice pre-treated with vehicle, quinpirole (0.01 mg/kg, i.p.) or tamoxifen (1 mg/kg, i.p.) in mice exposed to (A) vehicle (i.p., n=7-10) or (B) daidzein (100 mg/kg/day, i.p., n=8-9) during operant responding for cocaine. (PNG 457 kb)

High resolution image (TIF 78 kb)
