# Supplementary figures and images for: Daidzein modulates cocaine-reinforcing effects and cue-induced cocaine reinstatement in CD-1 male mice
Source: Psychopharmacology (Berl). 2021 Apr 11;238(7):1923–36. doi: 10.1007/s00213-021-05820-z (PMC8233246; doi:10.1007/s00213-021-05820-z)

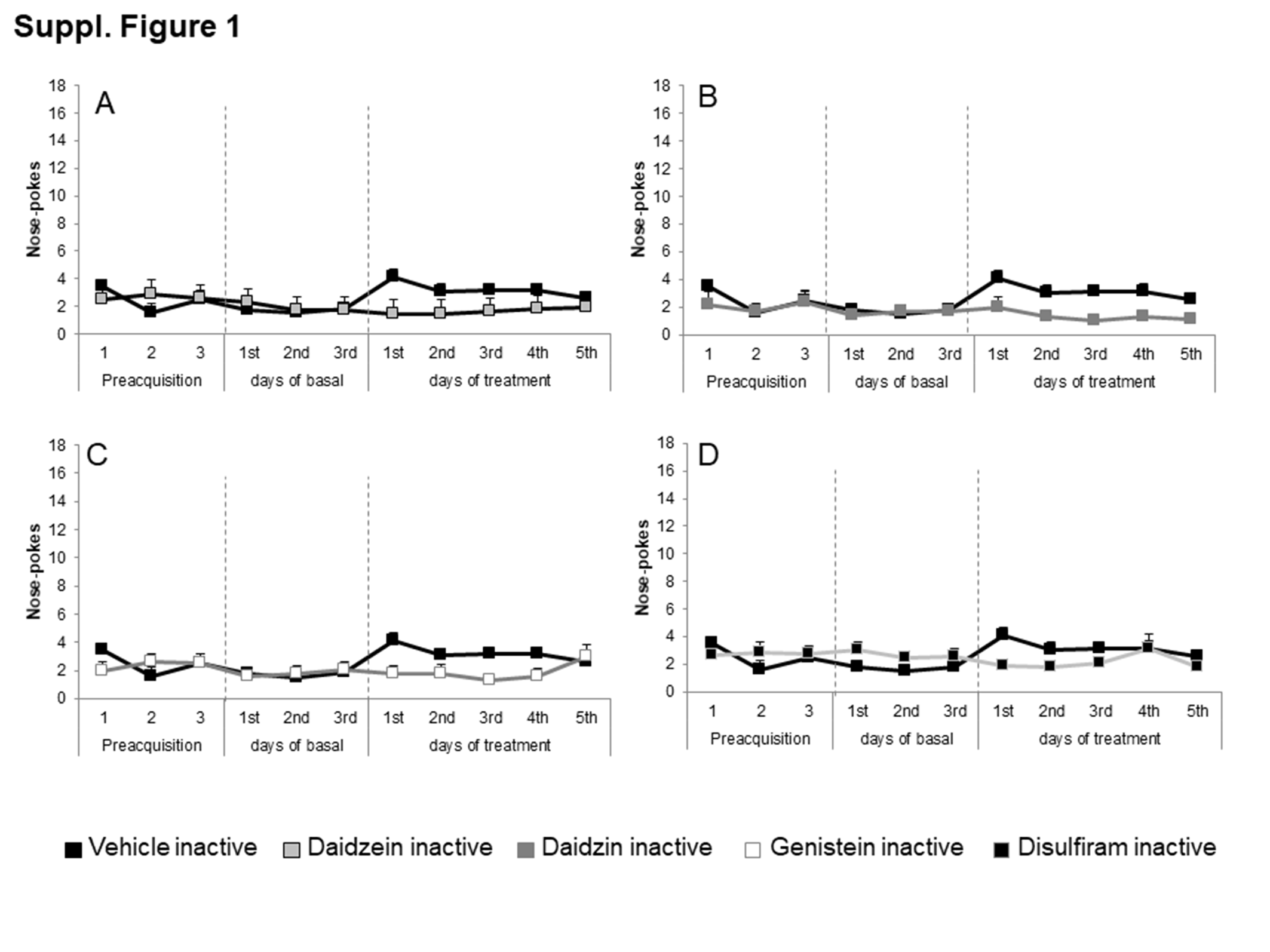

Supplement: Supplementary file 1 — Inactive nose-poke responses in mice treated with isoflavones during operant responding for cocaine. (A to D) Effects of the chronic treatment with vehicle (n=12) and daidzein (100 mg/kg/day, i.p., n=11) (A), daidzin (75 mg/kg/day, i.p., n=10) (B), genistein (100 mg/kg/day, i.p., n=10) (C) and disulfiram (75 mg/kg/day, i.p., n=9) (D) on the inactive nose-poke responding during cocaine self-administration. Results are expressed as average + SEM (PNG 550 kb) [file 213_2021_5820_Fig6_ESM.png]

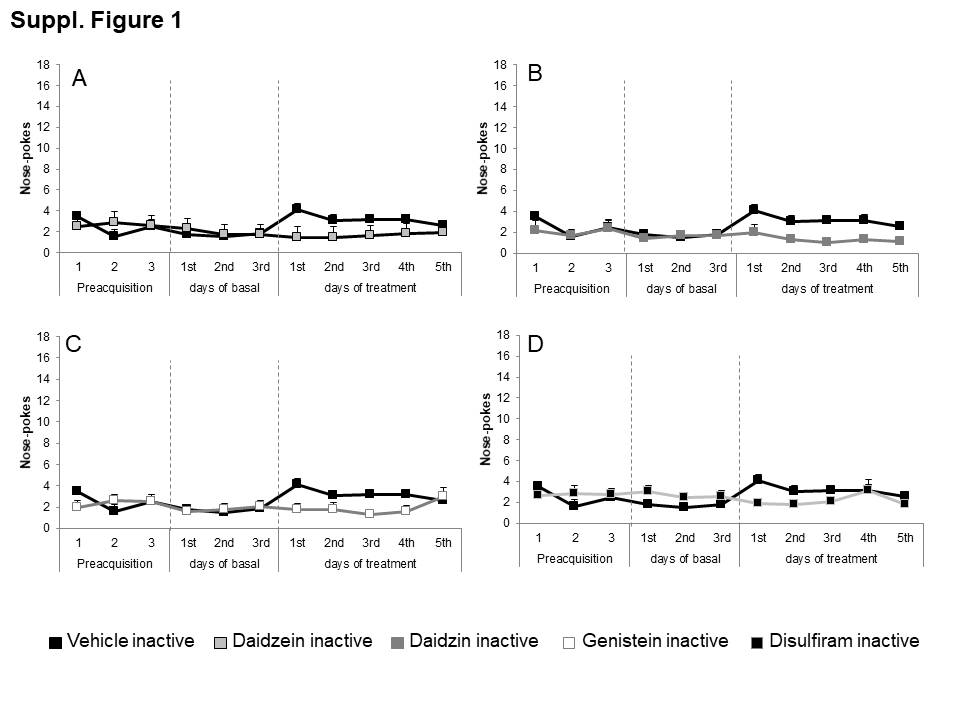

Supplement: Supplementary file 2 — High resolution image (TIF 85 kb) [file 213_2021_5820_MOESM1_ESM.tif]

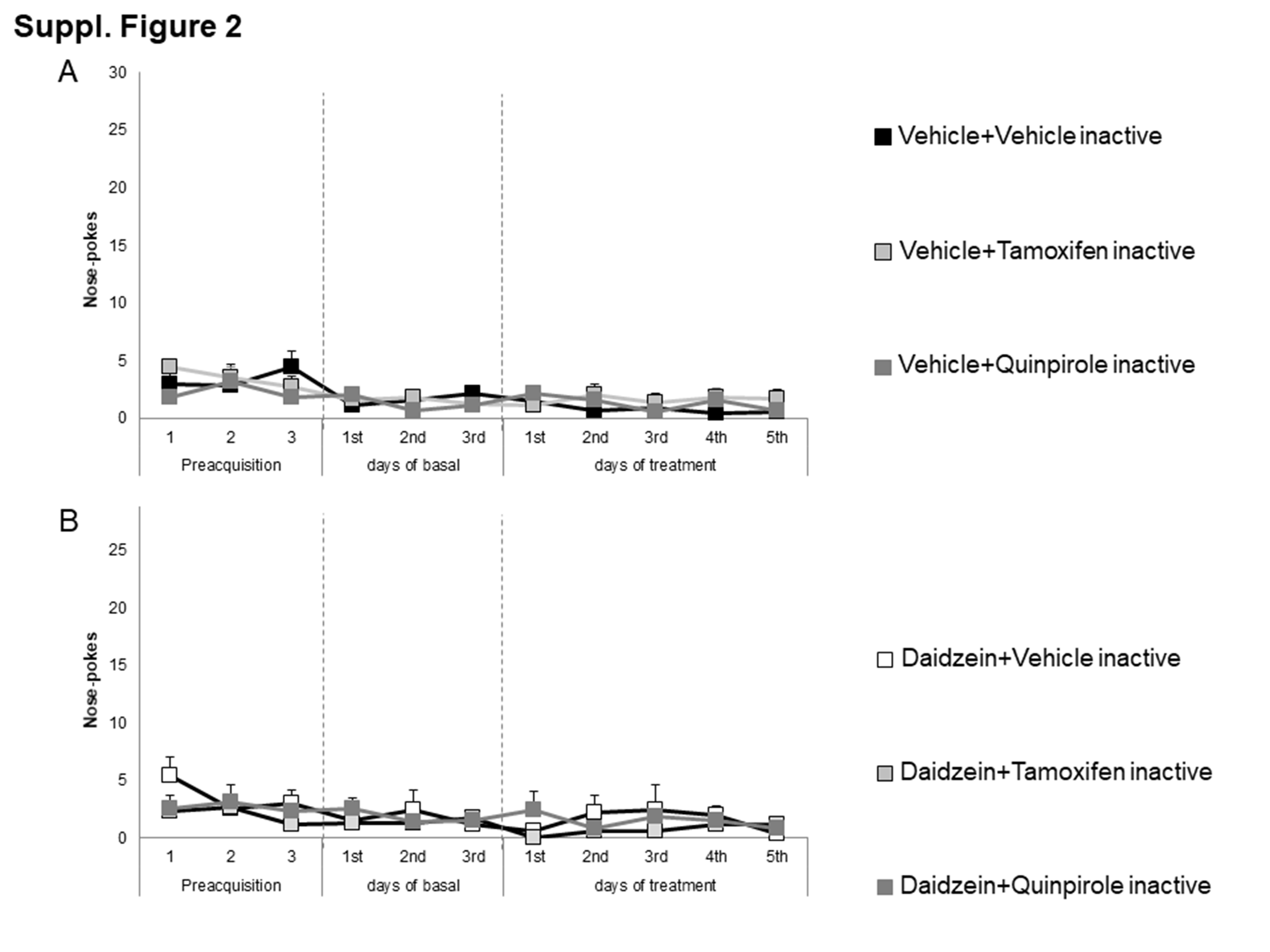

Supplement: Supplementary file 3 — Inactive nose-poke responses in mice pre-treated with vehicle, quinpirole (0.01 mg/kg, i.p.) or tamoxifen (1 mg/kg, i.p.) in mice exposed to (A) vehicle (i.p., n=7-10) or (B) daidzein (100 mg/kg/day, i.p., n=8-9) during operant responding for cocaine. (PNG 457 kb) [file 213_2021_5820_Fig7_ESM.png]

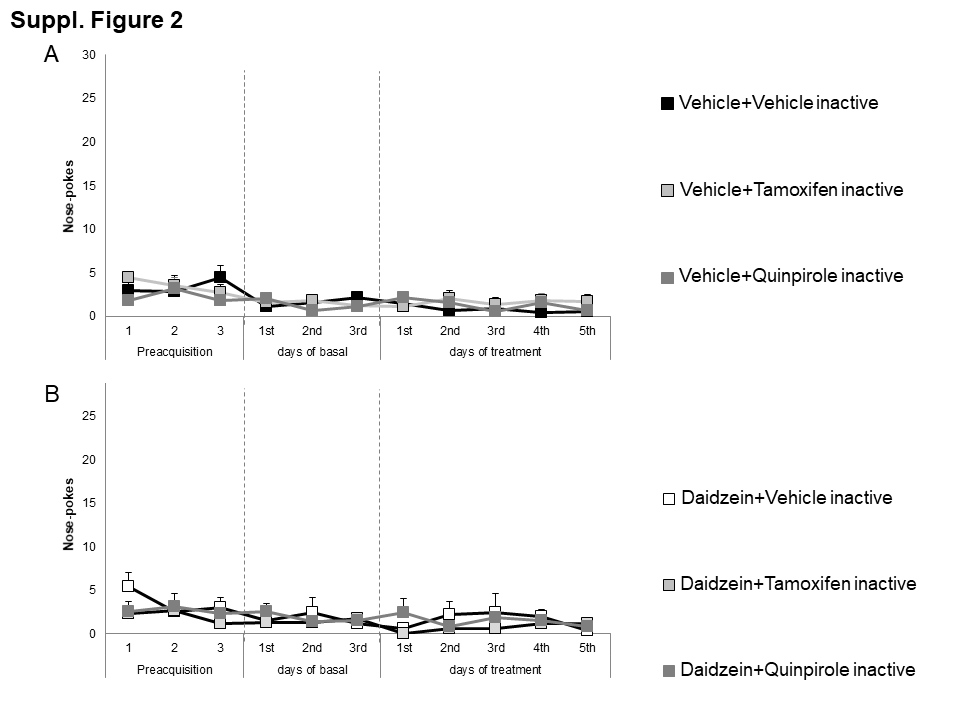

Supplement: Supplementary file 4 — High resolution image (TIF 78 kb) [file 213_2021_5820_MOESM2_ESM.tif]
